# The Effect P Additive on the CeZrAl Support Properties and the Activity of the Pd Catalysts in Propane Oxidation

**DOI:** 10.3390/ma17051003

**Published:** 2024-02-22

**Authors:** Feng Feng, Hong Li, Xingxia Yang, Chengxiong Wang, Yunkun Zhao, Hua Wang, Junchen Du

**Affiliations:** 1 Faculty of Metallurgical and Energy Engineering, Kunming University of Science and Technology, Kunming 650093, China; ff@spmcatalyst.com (F.F.); wanghua65@163.com (H.W.); 2 State-Local Joint Engineering Research Center of Precious Metal Catalytic Technology and Application, Kunming Sino-Platinum Metals Catalysts Co., Ltd., Kunming 650106, China; chengxiong.wang@spmcatalyst.com (C.W.); yk.zhao@spmcatalyst.com (Y.Z.); 3 Yunnan Precious Metal Laboratory Co., Ltd., Kunming 650100, China; lihong121727@163.com; 4 State Key Laboratory of Advanced Technologies for Comprehensive Utilization of Platinum Metals, Kunming Institute of Precious Metals, Kunming 650100, China

**Keywords:** phosphorus, CeO_2_-ZrO_2_-Al_2_O_3_, propane oxidation, palladium, support

## Abstract

The properties of a catalyst support are closely related to the catalyst activity, yet the focus is often placed on the active species, with little attention given to the support properties. In this work, we specifically investigated the changes in support properties after the addition of P, as well as their impact on catalyst activity when used for catalyst preparation. We prepared the CeO_2_-ZrO_2_-P_2_O_5_-Al_2_O_3_ (CeZrPAl) composite oxides using the sol–gel, impregnation, and mechanical mixing methods, and characterized the support properties using techniques such as XRD, XPS, SEM-EDS, N_2_ adsorption–desorption, and Raman spectra. The results showed that the support prepared using the sol–gel method can exhibit a more stable phase structure, larger surface area, higher adsorption capacity for oxygen species, and greater oxygen storage capacity. The addition of an appropriate amount of P is necessary. On the one hand, the crystallization and growth of CePO_4_ can lead to a decrease in the Ce content in the cubic phase ceria–zirconia solid solution, resulting in a phase separation of the ceria–zirconia solid solution. On the other hand, CePO_4_ can lock some of the Ce^3+^/Ce^4+^ redox pairs, leading to a reduction in the adsorption of oxygen species and a decrease in the oxygen storage capacity of the CeZrPAl composite oxides. The research results indicated that the optimal P addition is 6 wt.% in the support. Therefore, we prepared a Pd/CeZrPAl catalyst using CeZrAl with 6 wt.% P_2_O_5_ as the support and conducted the catalytic oxidation of C_3_H_8_. Compared with the support without P added, the catalyst activity of the support loaded with P was significantly improved. The fresh and aged (1000 °C/5 h) catalysts decreased by 20 °C and 5 °C in T_50_ (C_3_H_8_ conversion temperature of 50%), and by 81 °C and 15 °C in T_90_ (C_3_H_8_ conversion temperature of 90%), respectively.

## 1. Introduction

C_3_H_8_ is a common hydrocarbon in automotive exhaust emissions, which causes atmospheric pollution. One of the most effective ways to reduce air pollution is the catalytic combustion of hydrocarbons [1]. As reported, Pd is the preferred active metal for C_3_H_8_ combustion [2,3]. In order to improve the performance of the catalyst, researchers have made many attempts. Many researchers mainly focus on the preparation of core–shell catalysts [4], adding additives [5], etc. Among them, adding additives can change the state of precious metals, the properties of supports, and the interaction strength between supports and precious metals, thereby altering the activation pathway of C_3_H_8_ and improving the activity of catalysts [6]. The ultimate goal of these catalysts research is to apply them in practice to reduce environmental pollution. Considering industrial applications, adding additives to catalysts is the easiest way to achieve the goal; therefore, it is very necessary to study the role of additives in catalysts. So far, various additives have been reported, such as metallic [7,8,9] and non-metallic additives [10,11,12,13].

Metallic additives have been extensively studied, including rare earth metals (La, Pr, Y) [8,14,15], alkaline earth metals (Mg, Ba, Ca, Sr) [16,17,18], etc. For example, Zhao et al. [15] reported that the introduction of a series of rare earth metal additives (M = La^3+^, Nd^3+^, Pr^3+/4+^, and Y^3+^) into CeO_2_-ZrO_2_-Al_2_O_3_ (abbreviated as CeZrAl) could increase the surface oxygen vacancies, with Pr especially showing the most significant promoting effect. CeZrPrAl with more surface oxygen vacancies can establish stronger electronic interactions with the loaded Pd species and stabilize the Pd species in an oxidized state and a smaller particle size, thereby obtaining more active centers and increasing catalyst activity.

Non-metallic additives are also gradually being studied, especially phosphorus. Compared to metallic elements, phosphorus (P) has rich valence electrons and high electronegativity, which has been proven to be a suitable choice for enhancing catalytic performance. There are many studies on the catalytic activity of propane and CH_4_ with the addition of phosphorus to Pd/Al_2_O_3_ [19,20,21]. It has been proven that the addition of P can generate Brønsted acid sites on the Al_2_O_3_ surface, which is mainly related to the generation of P-OH groups and the redistribution of oxygen and aluminum atoms [13,19,20]. The introduction of P results in defects and vacancy in Al_2_O_3_, creating a coordination of unsaturated penta-coordinated aluminum sites. Thus, it increases the ability of Al_2_O_3_ to anchor active metal particles, preventing the loss of active ingredients [22]. In addition, the introduction of P can also adjust the morphology and thickness of γ-Al_2_O_3_ nanosheets [23]. Moreover, the presence of P in Al_2_O_3_ helps to achieve the optimal Pd^2+^/Pd^0^ molar ratio, reduce the adsorption of water by active sites on the catalyst through the rehydration of P-O groups, and control the redox properties of surface oxygen and hydroxyl groups, which ensure high activity, water resistance, and stability of the catalyst [21,24]. In summary, the increase in catalyst activity is not only due to the influence of additives on precious metals, but also due to the influence of additives on the support. The influence of additives on the support is also crucial, and it should be paid more attention to.

CeZrAl material is considered an excellent support material with a good oxygen storage capacity and resistance to hydrothermal aging. Du et al. [25] added P to CeZrAl and studied its effect on the combustion activity of CH_4_. The studies showed that the addition of P effectively regulates the surface Pd^2+^/(Pd^2+^ + Pd^0^) ratio, thereby effectively improving the catalytic activity of the catalyst for CH_4_. Unfortunately, they did not study the impact of P on the support, which limits our understanding of that. Therefore, this work focuses on the influence of the addition of P to a support. This study contributes to understanding the impact of additives on supports and provides a theoretical basis for the selection of additives for propane oxidation.

## 2. Materials and Methods

### 2.1. Materials

Al(NO_3_)_3_·9H_2_O (99.99%) and NH_3_·H_2_O (25~28%) were purchased from Xilong chemical Co., Ltd. (Shantou, China). (NH_4_)_2_HPO_4_ (99.99%) was obtained from Tianjin Fengchuan chemical reagent technology Co., Ltd. (Tianjin, China). Ce(NO_3_)_3_·6H_2_O (99.99%) and Zr(NO_3_)_4_·5H_2_O (99.99%) were purchased from West Asia chemical technology (Shandong) Co., Ltd. (Linyi, China) Pd(NO_3_)_2_ (24% Pd content) was obtained from Sino-Platinum Metals Co., Ltd. (Kunming, China). All chemicals were used as received without purification.

### 2.2. Synthesis of CeZrPAl Support Materials

#### 2.2.1. Synthesis of CeZrPAl with Different Preparation Methods

This work employed three different methods (sol–gel, impregnation, and mechanical mixing) to prepare CeZrPAl composite oxide materials. The composition of the materials is as follows: 74 wt.% Al_2_O_3_, 20 wt.% CeO_2_-ZrO_2_ (Ce:Zr = 1:1, molar ratio), and 6 wt.% P_2_O_5_.

The sol–gel method: Firstly, Al(NO_3_)_3_, Ce(NO_3_)_3_, and Zr(NO_3_)_4_ were dissolved in deionized water to prepare a mixed solution. A mixture of NH_3_·H_2_O and (NH_4_)_2_HPO_4_ in solution was slowly added dropwise into the mixed solution of Al(NO_3_)_3_, Ce(NO_3_)_3_, and Zr(NO_3_)_4_ while stirring continuously. Then, NH_3_·H_2_O was further added to adjust the pH to 6.5. After stirring for 1 h, the solution was left to stand at room temperature for 24 h. The resulting mixture was dried in a 100 °C oven until there was no change in weight. It was then calcined in a muffle furnace at 500 °C for 3 h to obtain fresh CeZrPAl composite oxides, denoted as CeZrPAl-G-F. Aging CeZrPAl composite oxides were obtained through further calcination of fresh CeZrPAl composite oxides in a muffle furnace at 1000 °C for 5 h, denoted as CeZrPAl-G-A.

The impregnation method: The mixed solution used in the sol–gel method was replaced with an aluminum nitrate solution, while other steps remained the same to prepare P-Al_2_O_3_ samples. Then, the P-Al_2_O_3_ was added to a mixed solution of Ce(NO_3_)_3_ and Zr(NO_3_)_4_, followed by continuous stirring for 6 h for impregnation. After standing at room temperature for 24 h, The sample was dried in a 100 °C oven until a constant weight was obtained. It was then calcined in a muffle furnace at 500 °C for 3 h to obtain fresh CeZrPAl composite oxides, denoted as CeZrPAl-I-F. Aging CeZrPAl composite oxides were obtained through further calcination of fresh CeZrPAl composite oxides in a muffle furnace at 1000 °C for 5 h, denoted as CeZrPAl-I-A.

The mechanical mixing method: Ce(NO_3_)_3_ and Zr(NO_3_)_4_ were dissolved in deionized water to prepare a mixed solution. NH_3_·H_2_O was added dropwise into the mixed solution while stirring until the pH reached 6.5. After stirring for 1 h, the solution was left to stand at room temperature for 24 h. The resulting mixture was dried in a 100 °C oven until a constant weight was obtained. Then, the mixture was placed in a muffle furnace and calcined at 500 °C to obtain a CeO_2_-ZrO_2_ sample. The P-Al_2_O_3_ prepared by the sol–gel method and CeO_2_-ZrO_2_ were mechanically mixed according to the stoichiometric ratio to obtain fresh CeZrPAl composite oxides, which were labeled CeZrPAl-M-F. Aging CeZrPAl composite oxides were obtained through further calcination of fresh CeZrPAl composite oxides in a muffle furnace at 1000 °C for 5 h, denoted as CeZrPAl-M-A.

#### 2.2.2. Synthesis of CeZrPAl with Different Phosphorus Contents

The preparation process of the CeZrPAl composite oxides with different P contents was the same as the sol–gel method. By adding different amounts of (NH_4_)_2_HPO_4_, fresh CeZrPAl composite oxides with different P contents (P_2_O_5_ contents of 0 wt.%, 2 wt.%, 6 wt.%, 10 wt.%) could be obtained, designated as CeZrAl-F, 2CeZrPAl-F, 6CeZrPAl-F, and 10CeZrPAl-F, respectively. The aged CeZrPAl composite oxides with different P contents were obtained after the fresh sample was calcined at 1000 °C for 5 h in a muffle furnace, which were designated as CeZrAl-A, 2CeZrPAl-A, 6CeZrPAl-A, and 10CeZrPAl-A.

#### 2.2.3. Synthesis of Pd/CeZrPAl and Pd/CeZrAl Catalysts

Pd/CeZrPAl and Pd/CeZrAl catalysts were prepared through impregnation using 6CeZrPAl and CeZrAl as supports. A Pd(NO_3_)_2_ precursor was prepared by adding an appropriate amount of deionized water to the Pd(NO_3_)_2_ solution and stirring for 30 min. Then, the palladium precursor solution of 1 wt.% Pd in catalysts was added to the supports, followed by continuous stirring for 6 h. After overnight impregnation at room temperature, the samples were dried in a 100 °C oven for 12 h and then calcined in a muffle furnace at 550 °C for 3 h. The obtained catalysts were named Pd/CeZrAl-F and Pd/6CeZrPAl-F. The aged catalysts were obtained after the fresh catalysts were calcined at 1000 °C for 5 h in a muffle furnace, which were designated as Pd/CeZrAl-A and Pd/6CeZrPAl-A.

### 2.3. Characterizations of CeZrPAl Support Materials

Low-temperature N_2_ adsorption–desorption experiments were conducted using a Quantachrome NOVA 2000e physical analyzer from Boynton Beach, FL, USA. The samples were pretreated at 200 °C under vacuum for 15 min and then subjected to adsorption–desorption at −196 °C using high-purity N_2_. The obtained data were calculated using the BET, t-Plot, and BJH methods to obtain the specific surface area, pore volume, and pore size distribution information of samples.

X-ray diffraction (XRD) was carried out using a Rigaku Smart Lab X-ray diffractometer from Tokyo, Japan. The analysis conditions were as follows: operating voltage of 40 kV, operating current of 80 mA, Cu Kα radiation source (λ = 1.5406 Å), scanning range of 2*θ* = 20°–80°, scanning step size of 0.02°, and scanning speed of 10°/min.

X-ray photoelectron spectroscopy (XPS) measurement was performed using a Thermo Scientific Kα X-ray photoelectron spectrometer from Waltham, MA, USA, using Al X-ray as its monochromatic source. The data were collected under the following conditions: vacuum level: 2 × 10^−7^ mbar, X-ray source: Al Kα (1486.6 eV), power: 72 W, and spot size: 400 μm. The XPS spectrum was corrected using surface contamination C1s (284.8 eV) as the standard.

Raman spectra were employed to analyze phase composition and structural analysis of the samples. The test was carried out using a Renishaw in Via confocal micro-Raman spectrometer from Renishaw, Gloucestershire, UK. The analysis conditions were as follows: excitation light source at 514.5 nm, spectral repeatability ≤ ±0.3 cm^−1^, spectral resolution of 1 cm^−1^, and spectral scanning range of 100–1400 cm^−1^.

EDS was used to study the chemical composition in the scanning electron microscope (SEM) mode (SEM-EDS) with a Hitachi TM3000 microscope from Tokyo, Japan.

Oxygen storage capacity (OSC) was determined using a NETZSCH STA-449-F3 simultaneous thermal analyzer from NETZSCH, Serb, Germany. The sample weighing 100 mg was placed in an Al_2_O_3_ crucible and heated from room temperature to 550 °C at a heating rate of 10 °C/min under a 10% O_2_/N_2_ atmosphere, and it was stabilized at 550 °C for 30 min for pretreatment. After pretreatment, the sample was cooled in an N_2_ atmosphere to 50 °C and kept for 45 min. Then, the sample was heated from 50 °C to 550 °C at a heating rate of 10 °C/min under a 10% H_2_/Ar atmosphere for 60 min. Finally, the sample was cooled to room temperature under a N_2_ atmosphere. The mass change signal of the sample was recorded and was used to calculate the total oxygen storage capacity based on the percentage of mass loss during the 10 Vol.% H_2_/Ar reaction.

### 2.4. Evaluation of C_3_H_8_ Oxidation Activity

The performance evaluation of the catalysts for propane oxidation was performed on a self-built atmospheric pressure multifunctional micro-fixed bed reactor. Gas composition and content were conducted online using the multi-gas series Fourier transform infrared (FTIR) gas analyzer from MKS in Andover, MA, USA. Catalyst powders were pressed, ground, and sieved to select 40–60 mesh catalyst particles for catalytic activity evaluation. A 0.5 g catalyst sample was loaded into a stainless-steel reaction with an inner diameter of 5 mm. The gas required for reaction was introduced until gas equilibrium, and then the temperature was raised from 150 °C to 600 °C at a heating rate of 10 °C/min for catalyst pretreatment. Then, it was cooled down to 100 °C; the temperature was further raised to 600 °C at a heating rate of 10 °C/min for the oxidation of C_3_H_8_. The composition of the reaction gases was 1000 ppm C_3_H_8_, 6000 ppm O_2_, and N_2_ was used as a balance gas, with a space velocity of 96,000 mL‧min^−1^‧g^−1^. The conversion of C_3_H_8_ was calculated according to the following formula:

$$\alpha_{\left(C_{3}H_{8},\%\right)}=\frac{C_{3}H_{8,in}-C_{3}H_{8,out}}{C_{3}H_{8,in}}\times 100\%$$

C_3_H_8,in_ and C_3_H_8,out_ represent the initial and remaining concentrations of C_3_H_8_, respectively. T_50_ and T_90_ are used to indicate the catalytic activity of propane oxidation, where T_50_ represents the reaction temperature at which 50% propane is converted, and T_90_ represents the reaction temperature at which 90% propane is converted.

## 3. Results and Discussion

### 3.1. Properties of CeZrPAl Support Materials

#### 3.1.1. The Composition and Structure of CeZrPAl Supports

**Figure 1:**
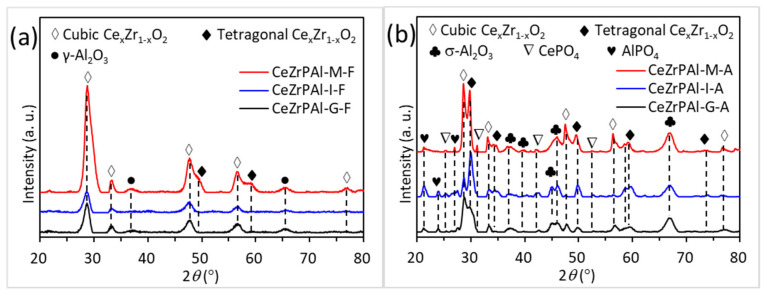
XRD patterns of CeZrPAl composite oxides prepared using different methods: (**a**) fresh samples and (**b**) aged samples.

The phase of the support greatly affects the activity of the catalyst; particularly, the stability of the phase is closely related to the stability of the catalyst. Therefore, we first studied the phase of the support before and after aging. Figure 1 shows the XRD patterns of fresh and aged CeZrPAl samples prepared using different methods. In Figure 1a, it can be seen that fresh CeZrPAl samples prepared using the sol–gel, impregnation, and mechanical mixing methods all exhibited diffraction peaks of the cubic-phase Ce_x_Zr_1−x_O_2_ solid solution at 2θ = 28.5°, 33.2°, 47.8°, 56.8°, and 76.8° (JCPDS#28-0271), and diffraction peaks of γ-Al_2_O_3_ at 2θ = 37.0° and 66.5° (JCPDS#47-1308). In addition, the CeZrPAl sample prepared using the mechanical mixing method also exhibited additional diffraction peaks of the tetragonal phase Ce_x_Zr_1−x_O_2_ at 2θ = 29.7°, 49.6°, and 59.7° (JCPDS#38-1437), indicating the presence of both cubic and tetragonal phases of a Ce_x_Zr_1−x_O_2_ solid solution in the CeZrPAl sample prepared using the mechanical mixing method. It is very obvious that the diffraction peak at 2θ = 28.5° of the support prepared using mechanical mixing was significantly stronger than that of the other two supports. This might have contributed to the larger grain size of CeO_2_-ZrO_2_ as raw materials in the mechanical mixing method. Compared with the CeZrPAl sample prepared using the mechanical mixing method, the diffraction peak intensity of the Ce_x_Zr_1−x_O_2_ solid solution and γ- Al_2_O_3_ was obviously weak in the CeZrPAl sample prepared using the gel method and impregnation method. The reason may be that Ce_x_Zr_1−x_O_2_ was highly dispersed in Al_2_O_3_ in the CeZrPAl samples prepared using the sol–gel method and impregnation method, while there was almost no interaction between Ce_x_Zr_1−x_O_2_ and Al_2_O_3_ in the CeZrPAl samples prepared using the mechanical mixing method. 

In Figure 1b, it can be seen that after aging, the diffraction peaks of γ-Al_2_O_3_ disappear, and new diffraction peaks of δ-Al_2_O_3_ appear at 2θ = 36.7°, 39.5°, 45.6°, 60.1°, and 67.0° (JCPDS#46-1133), indicating that γ- Al_2_O_3_ completely transforms into δ- Al_2_O_3_ after aging. The Ce_x_Zr_1−x_O_2_ solid solution in the aged CeZrPAl samples undergoes significant phase separation, with the coexistence of cubic and tetragonal phases of a Ce_x_Zr_1−x_O_2_ solid solution. Among them, the CeZrPAl samples prepared using the sol-gel and mechanical mixing methods mainly contain the cubic phase, while the CeZrPAl sample prepared using the impregnation method mainly contains the tetragonal phase. The intensities of the diffraction peaks of the cubic and tetragonal phases of the Ce_x_Zr_1−x_O_2_ solid solution are stronger in the CeZrPAl samples prepared using the impregnation and mechanical mixing methods compared to the CeZrPAl samples prepared using the sol–gel method, indicating that the impregnation and mechanical mixing methods result in larger crystal grains and a more severe phase separation of the Ce_x_Zr_1−x_O_2_ solid solution. After aging, the CeZrPAl samples exhibit diffraction peaks of AlPO_4_ at 2θ = 21.2°, 23.8°, and 27.0° (JCPDS#20-0045), and CePO_4_ at 2θ = 25.3°, 31.2°, 42.0°, and 52.8° (JCPDS#04-0632), indicating that P enters the lattice of the Al_2_O_3_ and Ce_x_Zr_1−x_O_2_ solid solutions to react with the Al_2_O_3_ and Ce_x_Zr_1−x_O_2_ solid solution to form AlPO_4_ and CePO_4_. No diffraction peaks of AlPO_4_ and CePO_4_ are observed in the fresh CeZrPAl samples, which can be attributed to the highly dispersed amorphous AlPO_4_ and CePO_4_ in the samples after calcinating at a low temperature (550 °C), while AlPO_4_ and CePO_4_ gradually crystallize and grow with increasing calcination temperature [10,12]. 

Figure S1 shows Raman spectra of the aged CeZrPAl composite oxides prepared using different methods. From the figure, it can be observed that all CeZrPAl samples exhibit five Raman peaks at 255 cm^−1^, 313 cm^−1^, 470 cm^−1^, 625 cm^−1^, and 972 cm^−1^. The peak at 470 cm^−1^ corresponds to the F_2g_ Raman-active mode of the cubic-phase Ce_x_Zr_1−x_O_2_ solid solution, attributed to the symmetric stretching mode of O atoms around Ce^4+^ ions [26,27]. The peaks at 255 cm^−1^, 313 cm^−1^, and 625 cm^−1^ correspond to the Raman-active modes of the tetragonal-phase Ce_x_Zr_1−x_O_2_ solid solution [28,29,30]. The peak at 972 cm^−1^ corresponds to the Raman-active mode of monoclinic CePO_4_ [31,32]. The presence of Raman-active modes of both cubic and tetragonal phases of the Ce_x_Zr_1−x_O_2_ solid solution in the aged samples indicates the coexistence of these phases. The CeZrPAl samples prepared using the sol–gel method and mechanical mixing method mainly exhibit Raman-active peaks at 470 cm^−1^, indicating the predominant presence of the cubic-phase Ce_x_Zr_1−x_O_2_ solid solution in the aged samples. On the other hand, the CeZrPAl samples prepared using the impregnation method mainly exhibit a Raman peak at 255 cm^−1^, indicating the predominant presence of the tetragonal-phase Ce_x_Zr_1−x_O_2_ solid solution in the aged samples. The Raman results are consistent with the XRD results. 

#### 3.1.2. Texture Properties of CeZrPAl Supports

**Figure 2:**
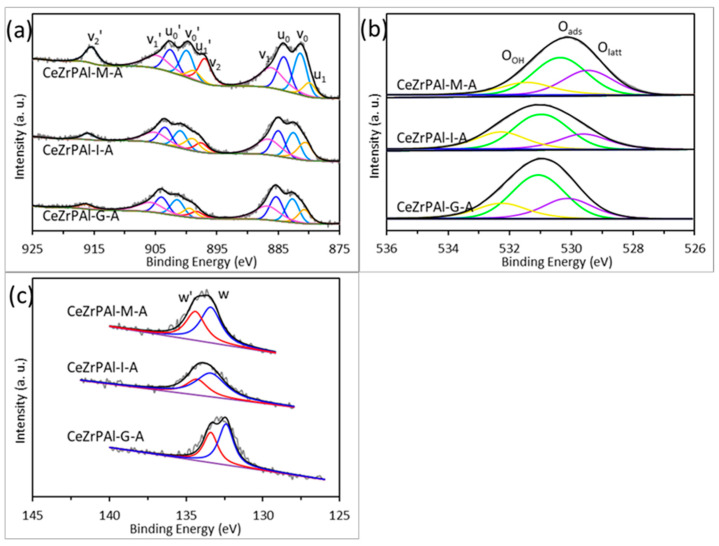
XPS spectra of CeZrPAl composite oxides prepared with different methods after aging (1000 °C): (**a**) Ce 3d, (**b**) O 1s, and (**c**) P 2p.

The microstructural properties of a material play a crucial role in determining its performance. Therefore, we investigated the properties of the material from a microscopic perspective. Figure 2a–c show the Ce 3d, O 1s, and P 2p XPS spectra of CeZrPAl composite oxides prepared using different methods after aging at 1000 °C, respectively. Table 1 summarizes the XPS data of aged CeZrPAl composite oxides prepared using different methods. From Figure 2a, it can be observed that the Ce 3d XPS spectrum contains ten characteristic peaks, where v_0_, v_1_, v_2_, v_0_′, v_1_′, and v_2_′ correspond to the characteristic peaks of Ce^4+^ 3d5/2, and u_0_, u_1_, u_0_′, and u_1_′ correspond to the characteristic peaks of Ce^3+^ 3d5/2 [33,34]. In Figure 2b, it can be seen that the O 1s XPS spectrum contains three characteristic peaks, where O_latt_ corresponds to the lattice oxygen on the surface, O_ads_ corresponds to the adsorbed oxygen species (e.g., O^2−^, O^2−^_2_, O^−^), and O_OH_ corresponds to the adsorbed water on the surface [14,35]. In Figure 2c, the P 2p peak contains two characteristic peaks, P 2p1/2 and P 2p3/2. The separation of binding energies between the two peaks is approximately 1.0 eV, consistent with that reported in [36]. Compared to the mechanical mixing method, it can be seen that the Ce 3d, O 1s, and P 2p spectra of CeZrPAl samples prepared using the sol–gel and impregnation methods exhibit significant shifts towards higher binding energies in Figure 2a–c. The results indicate that the electron-donating ability of P in the supports is stronger in the CeZrPAl samples prepared using the sol–gel and impregnation methods, which increases the covalent component of metal–oxygen and, thus, achieves a decreased Fermi level and acidity [37,38].

Table 1 shows the relative percentages of Ce^3+^ and O_ads_ on the surface of the CeZrPAl samples, which were calculated based on the fitting peak area ratios Ce^3+^/(Ce^3+^ + Ce^4+^) and O_ads_/(O_latt_ + O_ads_ + O_OH_), respectively. According to Table 1, the surface Ce^3+^ content of the CeZrPAl samples prepared using the sol–gel method, impregnation method, and mechanical mixing method is 36.7%, 41.4%, and 30.3%, respectively, while the O_ads_ content is 54.4%, 52.0%, and 49.6%, respectively. Compared with the mechanical mixing method, the content of Ce^3+^ on the surface of CeZrPAl samples prepared using the sol–gel method and impregnation method is higher, indicating that there are more oxygen vacancies in CeZrPAl samples prepared through the sol–gel method and impregnation method [39]. A high concentration of surface oxygen vacancies facilitates the formation of weakly adsorbed oxygen species and chemisorbed oxygen [40,41]. Therefore, the CeZrPAl samples prepared using the sol–gel and impregnation methods presented higher O_ads_ content compared to those prepared using the mechanical mixing method. In comparison to the sol–gel method, the CeZrPAl samples prepared using the impregnation method had a higher surface Ce^3+^ content but a relatively lower O_ads_ content. This is because the formation of CePO_4_ in the CeZrPAl samples prepared using the impregnation method leads to the reduction of Ce (IV) to Ce (III), resulting in a higher surface Ce^3+^ content [31]. However, CePO_4_ is a highly stable Ce (III) phosphate that cannot be oxidized or reduced. The formation of CePO_4_ locks some Ce^3+^/Ce^4+^ redox pairs, preventing the formation of oxygen vacancies in CeZrPAl samples and reducing the content of surface-adsorbed oxygen [32,42]. Table S1 shows the surface P content measured on SEM-EDS. From the table, it can be seen that the amount of P added to the support is the same for different preparation methods, but the surface P content measured is different. This is mainly related to the catalyst preparation method. As we know, the impregnation and mechanical mixing methods used the prepared P-Al_2_O_3_ as raw material, so the P content on the surface was slightly less than that of the sol–gel method. In addition, the interaction between P and Ce in the support prepared with the sol–gel method was stronger than the other two methods. This result is consistent with the XPS results.

**Figure 3:**
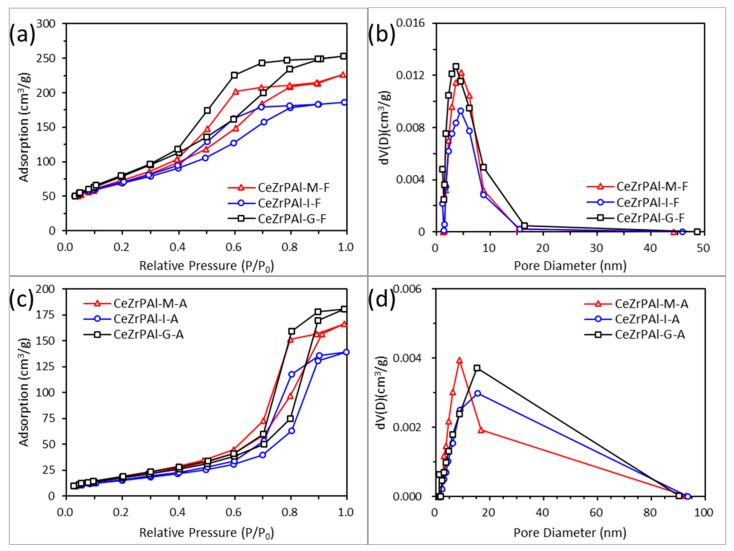
The adsorption and desorption isotherms ((**a**) fresh and (**c**) aged samples) and pore size distribution ((**b**) fresh and (**d**) aged samples) of CeZrPAl composite oxides prepared using different methods.

The structural parameters of a support before and after aging can indicate the stability of the support, determining the quality of the support. Figure 3 shows the adsorption-desorption isotherms of fresh and aged CeZrPAl samples prepared using different methods. From Figure 3a,b, it can be observed that the CeZrPAl samples prepared using different methods exhibit similar adsorption isotherms and hysteresis loops before and after aging, indicating that the preparation method has no significant effect on the pore type of the CeZrPAl samples. According to the IUPAC classification, both fresh and aged CeZrPAl samples exhibit type IV isotherms [43]. The fresh CeZrPAl sample exhibits an H2 hysteresis loop and has a pore structure characterized by cylindrical and ink bottle-shaped mixed pores, where the pore openings are similar in size to the pore channels/cavities, facilitating mass and heat transfer during reactions [44]. After high-temperature aging, the CeZrPAl samples undergo a transition from an H2 hysteresis loop to an H3 hysteresis loop, and the pore structure changes from cylindrical and ink bottle-shaped mixed pores to slit-shaped pores. The slit pores might be narrow gaps formed through the sintering and aggregation of particles during the samples’ aging [43].

Figure 3b,d show the pore size distribution curves of fresh and aged CeZrPAl samples prepared using different methods. Although the preparation method has no significant effect on the pore type of the CeZrPAl samples, there are distinct differences in pore-size distribution. In Figure 3b, it can be seen that the pore size distribution of fresh CeZrPAl samples prepared using different methods is within the range of 2–16 nm, indicating the presence of mesopores in the samples. Compared with the sample prepared using the impregnation method and mechanical mixing method, the CeZrPAl samples prepared using the sol–gel method exhibit a shift towards smaller pores, with a portion of the pore size distribution being in the range of 0–2 nm, indicating the presence of some micropores in the sample. The formation of micropores might be due to the decomposition of ammonium salts during the calcination process of phosphorus-containing samples [45]. The more the micropores present, the larger the specific surface area and pore volume. In summary, the CeZrPAl samples prepared using the sol–gel method have a larger specific surface area and pore volume. After aging, significant sintering and agglomeration occur in the samples, causing the small pores to gradually enlarge and become macropores. The pore size distribution of aged samples shifts towards larger pores, the width of the pore size distribution increases, and the range of pore sizes expands from 2–16 nm to 2–100 nm, which indicates that the pore structure of the support increases after adding P. This might be attributed to the chemical reaction between the support materials and phosphorus that destroys or expands the size of the small pores [46]. The samples undergo a transition from a mesoporous pore structure to a meso-macroporous composite pore structure.

Table 2 shows the specific surface area and pore structure parameters of fresh and aged CeZrPAl samples prepared using different methods. From the table, it can be seen that in the fresh samples, CeZrPAl samples prepared using the sol–gel method have the highest specific surface area of 323 m^2^‧g^−1^, while the specific surface areas of CeZrPAl samples prepared using the impregnation method and mechanical mixing method are relatively lower, at 217 m^2^‧g^−1^ and 255 m^2^‧g^−1^, respectively. After aging, the specific surface area and the pore volume of the CeZrPAl samples decrease sharply, and the average pore size increases. After aging, the CeZrPAl samples prepared using the sol–gel method and mechanical mixing method exhibit relatively large specific surface areas of 88 m^2^‧g^−1^ and 95 m^2^‧g^−1^, respectively. Meanwhile the specific surface area of the CeZrPAl samples prepared using the impregnation method is only 69 m^2^‧g^−1^, indicating the poorest stability. Compared with CeZrPAl samples prepared using the mechanical mixing method, the CeZrPAl samples prepared using the sol–gel method exhibit larger pore volumes before and after aging, indicating superior texture properties and thermal stability.

Compared with the mechanical mixing method, CeZrPAl samples prepared using the sol–gel and impregnation methods exhibit a significant increase in pore size. the reason might be that the CeZrPAl samples prepared using the sol–gel method are prone to a collapse of the small pore structure, leading to a significant increase in pore size after aging. For the CeZrPAl samples prepared using the impregnation method, the blockage of Al_2_O_3_ pores by Ce_x_Zr_1−x_O_2_ after aging leads to an increase in pore size.

#### 3.1.3. Oxygen Storage Capacity (OSC) of CeZrPAl Supports

The oxygen storage capacity of a material is an important indicator for determining the strength of the catalyst’s oxidation–reduction ability. OSC is generally classified into two categories: total OSC (TOSC) and dynamic OSC (DOSC). TOSC represents the total amount of transferable oxygen at a fixed temperature, including surface and bulk oxygen. DOSC represents the most active oxygen species and easily obtainable oxygen atoms, involving surface oxygen and oxygen vacancies [47]. The OSC in this study is actually DOSC. Table 3 shows the OSC of fresh and aged CeZrPAl samples prepared using different methods. From Table 3, it can be observed that the oxygen storage capacities of the CeZrPAl samples prepared using the sol–gel method, impregnation method, and mechanical mixing method before aging are 294 μmol/g, 306 μmol/g, and 212 μmol/g, and after aging, the values are 56 μmol/g, 50 μmol/g, and 37 μmol/g, respectively. Table S2 shows the oxygen storage capacity of the support prepared with different methods based on normalized surface area data, with a trend similar to that of OSC. Compared with the mechanical mixing method, the CeZrPAl samples prepared with the sol–gel method and impregnation method exhibit higher oxygen storage capacities both before and after aging. According to XPS results, this may be attributed to the introduction of Al^3+^ into the crystal lattice of the Ce_x_Zr_1−x_O_2_ solid solution in CeZrPAl samples prepared using the sol–gel and impregnation methods, causing lattice distortion and generating more oxygen vacancies and lattice defects in the Ce_x_Zr_1−x_O_2_ solid solution. As a result, the oxygen storage capacity of the CeZrPAl samples is enhanced [48,49].

In summary, the CeZrPAl composite oxide prepared using the sol–gel method has the best performance and is considered to be the best preparation method. Therefore, we chose this method as the method for preparing the support for the following study.

### 3.2. Effect of P Additive Amount on Supports’ Properties

The content of additives added has a significant impact on the performance of a support material, so we further studied the effect of the addition of P on the performance of the support material.

#### 3.2.1. Texture Properties of CeZrPAl Supports

**Figure 4:**
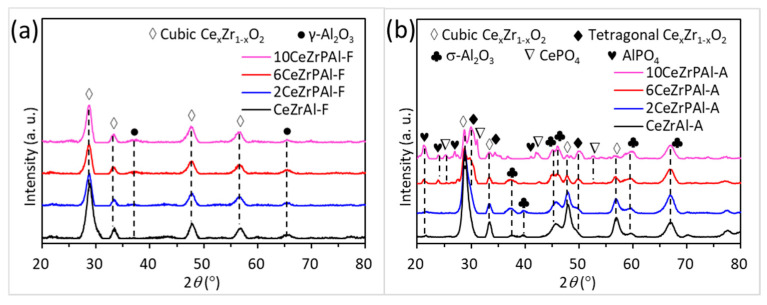
XRD patterns of CeZrPAl composite oxides with different P contents: (**a**) fresh samples and (**b**) aged samples.

Figure 4 shows the XRD patterns of fresh and aged CeZrPAl composite oxides with different P contents. From Figure 4a, it can be observed that all fresh CeZrPAl samples exhibit diffraction peaks at 2θ = 28.5°, 33.2°, 37.6°, 47.8°, 56.8°, and 76.8°, corresponding to the cubic phase of the Ce_x_Zr_1−x_O_2_ solid solution (JCPDS#28-0271), as well as diffraction peaks at 2θ = 37.6°, 43.2°, and 67.0°, corresponding to γ-Al_2_O_3_ (JCPDS#47-1308). With an increase in the P content, the intensity of the diffraction peaks of the cubic-phase Ce_x_Zr_1−x_O_2_ solid solution and γ-Al_2_O_3_ significantly decreases, indicating that the addition of P reduces the crystallinity of the Ce_x_Zr_1−x_O_2_ solid solution and γ-Al_2_O_3_ in the CeZrPAl samples [24]. In Figure 4b, it can be seen that after aging, the diffraction peak of γ-Al_2_O_3_ disappears at 2θ = 66.5°, and new diffraction peaks of δ-Al_2_O_3_ appear at 2θ = 36.7°, 39.5°, 45.6°, 60.1°, and 67.0° (JCPDS#46-1133), indicating a complete phase transformation of γ-Al_2_O_3_ to δ-Al_2_O_3_. The Ce_x_Zr_1−x_O_2_ solid solution in the aged CeZrAl and 2CeZrPAl samples still maintains a cubic phase structure, with only a significant increase in diffraction peak intensity. The result indicates that the Ce_x_Zr_1−x_O_2_ solid solution undergoes sintering during the aging process, leading to agglomeration and an increase in size. However, in the aged 6CeZrPAl and 10CeZrPAl samples, significant phase separation occurred in the Ce_x_Zr_1−x_O_2_ solid solution. Not only are there diffraction peaks of the cubic Ce_x_Zr_1−x_O_2_ solid solution at 2θ = 28.5°, 33.2°, 47.8°, 56.8°, and 76.8°, but there are also diffraction peaks of the tetragonal-phase Ce_x_Zr_1−x_O_2_ solid solution appearing at 2θ = 29.7°, 34.4°, 49.6°, 59.7°, and 73.7° (JCPDS#38-1437). The results indicate that phase separation of Ce_x_Zr_1−x_O_2_ occurs at high temperature when the P content in the CeZrPAl samples exceeds 6 wt.%. In the aged 6CeZrPAl and 10CeZrPAl samples, the diffraction peaks of AlPO_4_ appear at 2θ = 21.2°, 23.8°, and 27.1° (JCPDS#20-0045), and the diffraction peaks of CePO_4_ appear at 2θ = 25.3°, 31.2°, 42.0°, and 52.8° (JCPDS#04-0632). With an increase in the P content, the intensities of the diffraction peaks of AlPO_4_ and CePO_4_ significantly increase, indicating a higher content of AlPO_4_ and CePO_4_ in CeZrPAl samples with a higher P content. However, no diffraction peaks of AlPO_4_ and CePO_4_ are observed in the fresh and aged 2CeZrPAl samples. This might be because AlPO_4_ and CePO_4_ are highly dispersed in an amorphous form in fresh CeZrPAl samples, and with the increase in calcination temperature, AlPO_4_ and CePO_4_ gradually crystallize and grow. In addition, the phosphorus content is low in the 2CeZrPAl samples, and the content of AlPO_4_ and CePO_4_ in the aged sample is still below the detection limit of XRD; thus, no diffraction peaks of AlPO_4_ and CePO_4_ are observed.

Figure S2 shows the Raman spectra of CeZrPAl samples with different P contents after aging at 1000 °C. From the figure, it can be observed that all CeZrPAl samples exhibit a broad intense peak at 470 cm^−1^, which corresponds to the F_2g_ Raman-active mode of the cubic-phase cerium–zirconium solid solution and is attributed to the symmetric stretching mode of oxygen atoms around Ce^4+^ ions [26,27]. Compared to the CeZrAl sample, the 6CeZrPAl and 10CeZrPAl samples exhibit three weak shoulder peaks at 267 cm^−1^, 316 cm^−1^, and 634 cm^−1^, which correspond to the Raman-active modes of the tetragonal-phase Ce_x_Zr_1−x_O_2_ solid solution [28,29,30], indicating that when the phosphorus content in the aged CeZrPAl samples exceeds 6 wt.%, a phase separation of the Ce_x_Zr_1−x_O_2_ solid solution occurs under high-temperature conditions. The 6CeZrPAl and 10CeZrPAl samples exhibit a peak at 972 cm^−1^, corresponding to the Raman-active characteristic peak of monoclinic CePO_4_ [31,32], indicating the presence of CePO_4_ in the aged 6CeZrPAl and 10CeZrPAl samples. In addition, the 10CeZrPAl sample exhibits peaks at 1058 cm^−1^ and 1124 cm^−1^, corresponding to the Raman-active characteristic peaks of CePO_4_ and AlPO_4_, respectively [32,50], indicating the presence of CePO_4_ and AlPO_4_ in the aged 10CeZrPAl samples, which is consistent with the XRD characterization results.

In summary, with an increase in phosphorus content in the CeZrPAl aged samples, the intensity of the diffraction peaks of the cubic-phase Ce_x_Zr_1−x_O_2_ solid solution gradually decreases, while the intensity of the diffraction peaks of the tetragonal-phase Ce_x_Zr_1−x_O_2_ gradually increases. This might be because the higher P content in the CeZrPAl samples leads to the formation of more CePO_4_, and the crystallization of CePO_4_ at high temperatures reduces the Ce content in the cubic-phase Ce_x_Zr_1−x_O_2_ solid solution, which result in the phase separation of Ce_x_Zr_1−x_O_2_.

#### 3.2.2. Textural Properties of CeZrPAl Supports with Different P Contents

**Figure 5:**
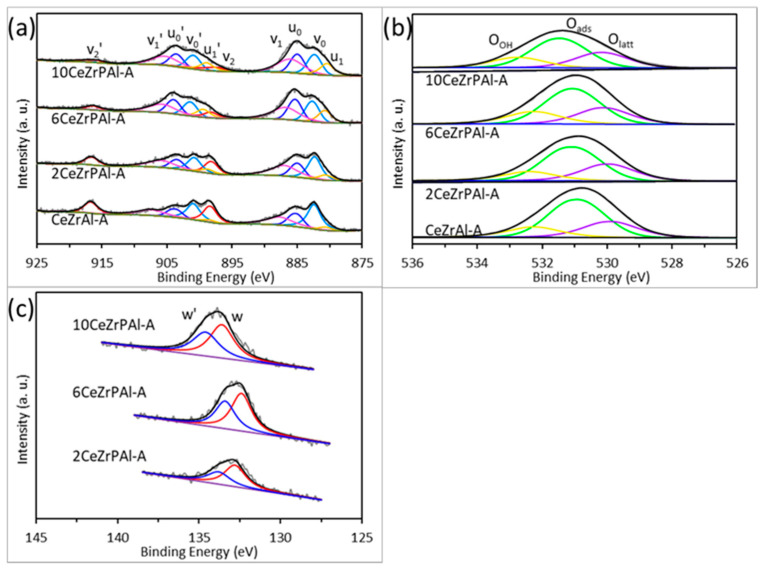
XPS spectra of (**a**) Ce 3d, (**b**) O 1s, and (**c**) P 2p of CeZrPAl composite oxides with different phosphorus contents after aging (1000 °C).

Figure 5a–c show the Ce 3d, O 1s, and P 2p XPS spectra of CeZrPAl composite oxides with different phosphorus contents after aging at 1000 °C, respectively. From Figure 5a, it can be observed that the Ce 3d XPS spectrum contains ten characteristic peaks, where v_0_, v_1_, v_2_, v_0_′, v_1_′, and v_2_′ correspond to the characteristic peaks of Ce^4+^ 3d5/2, and u_0_, u_1_, u_0_′, and u_1_′ correspond to the characteristic peaks of Ce^3+^ 3d5/2 [34,51]. In Figure 5b, it can be seen that the O 1s XPS spectrum contains three characteristic peaks, where O_latt_ corresponds to the lattice oxygen on the surface, O_ads_ corresponds to the adsorbed oxygen species (e.g., O^2−^, O^2−^_2_, O^−^), and O_OH_ corresponds to the adsorbed water on the surface [10,14]. In Figure 5c, the P 2p peak contains two characteristic peaks: P 2p1/2 and P 2p3/2. The separation of the binding energies between the two peaks is approximately 1.0 eV, which is consistent with the value reported in [36]. Compared with the CeZrAl sample, it can be observed that the Ce 3d*,* O 1s, and P 2p spectra of the 2CeZrPAl, 6CeZrPAl, and 10CeZrPAl samples gradually shift towards higher binding energies. As discussed earlier, it might be attributed to the increasement in the covalent composition of the metal–oxygen and the decrease in the Fermi level and acidity [38]. The result also indicates that there is an interaction between Ce and P, which becomes stronger with an increase in the P content [37]. Table 4 presents the relative percentage of surface Ce^3+^ and O_ads_ content in CeZrPAl samples, which were calculated based on the fitting peak area ratio of Ce^3+^/(Ce^3+^ + Ce^4+^) and O_ads_/(O_latt_ + O_ads_ + O_OH_). Table S3 shows the surface P content measured on SEM-EDS. From the table, it can be seen that no P was detected in the fresh and aged samples of CeZrAl and 2CeZrPAl. It might be because the CeZrAl was a blank sample and the P content added to 2CeZrPAl was relatively low and evenly distributed. It is interesting that the peak of P 2p can be detected through XPS but cannot be detected through EDS, which might be because of the lower detection limit of the instrument. As the amount of P added increased, the detected P content on the surface of the support gradually increased. This result is consistent with the XPS result.

From Table 4, it can be observed that the surface Ce^3+^ content of the CeZrAl, 2CeZrPAl, 6CeZrPAl, and 10CeZrPAl samples is 27.0%, 27.1%, 36.7%, and 38.8%, respectively, while the O_ads_ content is 58.1%, 56.2%, 54.4%, and 53.8%, respectively. With an increase in the P content, the content of Ce^3+^ in the CeZrPAl samples gradually increases, while the content of the surface-adsorbed oxygen species gradually decreases. This might be because the CeZrPAl samples with a higher P content have a higher content of CePO_4_. The generation of CePO_4_ leads to a reduction of Ce (IV) to Ce (III) in the CeZrPAl samples, which results in a gradual increase in the Ce^3+^ content [31]. The formation of Ce^3+^ species is closely related to the generation of oxygen vacancies (4Ce^4+^ + O^2−^ → 4Ce^4+^ + 2e^−^/δ + 0.5O_2_ → 2Ce^4+^ + 2Ce^3+^ + δ + 0.5O_2_, where δ represents oxygen vacancies). A high concentration Ce^3+^ species creates more oxygen vacancies, which can effectively absorb O_2_ and form more reactive oxygen species. However, CePO_4_ is a highly stable Ce (III) phosphate that cannot be oxidized or reduced, even under high-temperature and oxygen-rich conditions. Therefore, the formation of CePO_4_ locks in some Ce^3+^/Ce^4+^ redox pairs, resulting in the inability of Ce^3+^ in CePO_4_ to form oxygen vacancies. As a result, the CeZrPAl samples with a higher phosphorus content have fewer oxygen vacancies and a lower content of surface-adsorbed oxygen species [32,42].

Table 5 presents the specific surface area and pore structure parameters of fresh and aged CeZrPAl samples with different phosphorus contents. From the table, it can be observed that the specific surface area and pore volume of fresh CeZrPAl samples gradually increase, while the pore size decreases with an increase in phosphorus content. Particularly, the specific surface area of the 10CeZrPAl sample reaches a maximum of 341 m^2^/g. After aging, the specific surface area and pore volume of all CeZrPAl samples decrease sharply, and the pore size increases significantly. The changes in specific surface area, pore volume, and pore size of aged CeZrPAl samples do not show a consistent trend with increasing phosphorus content. Compared with the CeZrAl sample, the specific surface area of the 6CeZrPAl sample slightly increases, while the specific surface area of the 2CeZrPAl and 10CeZrPAl samples slightly decreases. The result indicates that doping an appropriate amount of phosphorus (6 wt.%) can improve the stability of CeZrPAl samples.

**Figure 6:**
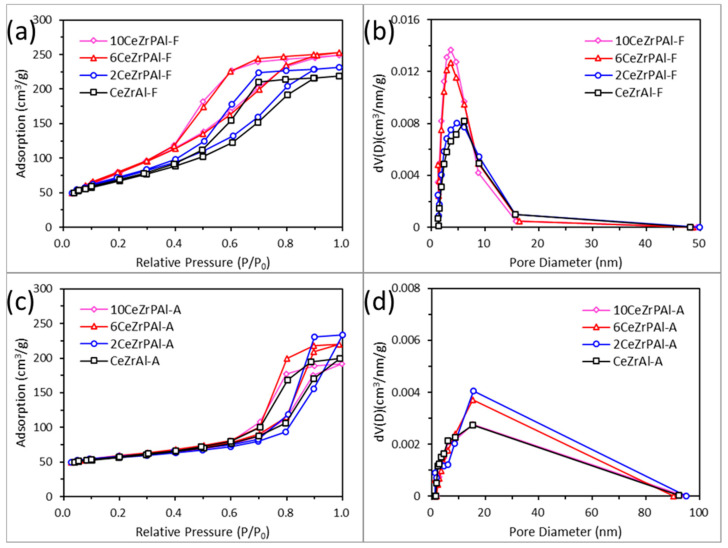
Adsorption and desorption isotherms ((**a**) fresh samples and (**c**) aged samples) and pore size distribution ((**b**) fresh samples and (**d**) aged samples) of CeZrPAl composite oxides with different P contents.

To further investigate the reasons behind these changes, Figure 6 shows the adsorption–desorption isotherms of fresh and aged CeZrPAl samples with different phosphorus contents. In Figure 6a,c, it can be seen that CeZrPAl samples with different phosphorus contents exhibit similar adsorption isotherms and hysteresis loops before and after aging, indicating that the phosphorus content has no significant effect on the pore type of CeZrPAl samples. According to the IUPAC classification, all CeZrPAl samples exhibit type IV isotherms before and after aging [43]. The fresh CeZrPAl samples exhibit an H2 hysteresis loop and have a pore structure characterized by cylindrical and ink bottle-shaped mixed pores, where the pore openings are similar in size to the pore channels, which facilitates mass and heat transfer during reactions [44]. After aging, the CeZrPAl sample undergoes a transition from an H2 hysteresis loop to an H3 hysteresis loop, and the pore structure changes from cylindrical and ink bottle-shaped mixed pores to slit-shaped pores. The slit pores are narrow gaps formed through the sintering and aggregation of particles, indicating that the CeZrPAl sample undergoes sintering and agglomeration during the aging process.

Figure 6b,d show the pore size distribution curves of fresh and aged CeZrPAl samples with different phosphorus contents. It can be observed that the pore size distribution of fresh CeZrPAl samples is within the range of 2–16 nm, indicating the presence of mesopores in the samples. With an increase in phosphorus content in the CeZrPAl samples, the pore size distribution gradually shifts towards smaller pores, with a portion of the pore size distribution in the range of 0–2 nm, indicating the presence of some micropores in the samples. The formation of micropores is due to the decomposition of ammonium salts during the calcination process of phosphorus-containing samples [45]. Therefore, as the phosphorus content increases, the number of micropores in the sample also increases, resulting in a larger specific surface area and pore volume of the sample. After aging, significant sintering and agglomeration occur in the samples, causing the small pores to gradually enlarge and become macropores. The pore size distribution of aged samples shifts towards larger pores, the width of the pore size distribution increases, and the range of pore sizes expands from 2–16 nm to 2–100 nm, which indicate that the pore structure of the support increases after adding P. This might be attributed to the chemical reaction between the support materials and phosphorus, which destroys or expands the size of the small pores [46]. The samples undergo a transition from a mesoporous pore structure to a meso-macroporous complex pore structure. Compared to the aged CeZrAl sample, the pore size distribution of the 6CeZrPAl and 10CeZrPAl samples is similar, while the 2CeZrPAl sample shows a slight shift towards larger pores. The result indicates that a lower phosphorus content had a negative impact on the pore structure of the CeZrPAl samples, and doping an appropriate amount of phosphorus was beneficial for stabilizing the pore structure of the CeZrPAl samples.

#### 3.2.3. Oxygen Storage Capacity (OSC) of CeZrPAl Supports with Different P Contents

The oxygen storage capacities of the CeZrPAl samples with different phosphorus contents was also investigated. Table 6 shows the OSC of fresh and aged CeZrPAl samples. From the table, it can be observed that the OSC of the CeZrAl, 2CeZrPAl, 6CeZrPAl, and 10CeZrPAl samples before and after aging are 313 μmol/g, 306 μmol/g, 294 μmol/g, 231 μmol/g, 125 μmol/g, 81 μmol/g, 56 μmol/g, and 63 μmol/g, respectively. With an increase in the phosphorus content, the OSC of the CeZrPAl samples decrease gradually before and after aging. As discussed in Section 3.1.3, the measurement used in this study was DOSC. Table S4 shows the oxygen storage capacity of the support with different levels of P addition based on normalized surface area data, with a trend similar to that of OSC. Based on the results of the XRD, Raman, and XPS analyses, this is because as the phosphorus content increases in the CeZrPAl samples, the content of CePO_4_ increases, and the formation of CePO_4_ locks some Ce^3+^/Ce^4+^ redox pairs, resulting in a gradual decrease in the oxygen storage capacity of the CeZrPAl samples [31,32].

#### 3.2.4. Activity of C_3_H_8_ Oxidation

**Figure 7:**
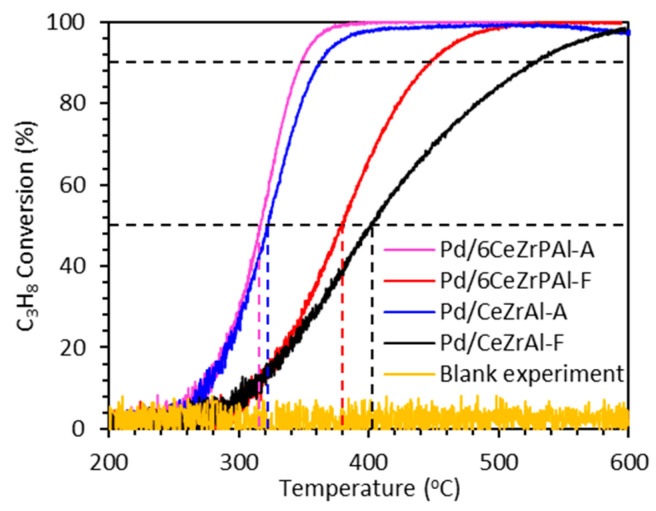
Conversion curves of propane under different catalysts.

The suitability of a catalyst support depends on the activity of the catalyst when the active species is loaded onto the support. Therefore, we prepared corresponding Pd-based catalysts using supports without and with P added, respectively. We evaluated their oxidation ability to C_3_H_8_ to assess the effect of the support on catalyst activity. As shown in Figure 7, we evaluated their oxidation ability towards C_3_H_8_ to assess the influence of support properties on the overall catalyst activity. Before the experiment, we conducted a blank experiment, and the results showed that C_3_H_8_ could not be oxidized when there was no catalyst in the reaction. According to industry standards, we measured the temperatures at which 50% (T_50_) and 90% (T_90_) conversion of C_3_H_8_ occurred. The T_50_ and T_90_ of the fresh Pd/6CeZrPAl-F catalyst were 380 °C and 447 °C, respectively, while the T_50_ and T_90_ of the aged catalyst were 317 °C and 348 °C, respectively. The T_50_ and T_90_ of the fresh sample without adding P (Pd/CeZrAl-F) were 400 °C and 528 °C, respectively, while the T_50_ and T_90_ of the aged catalyst were 323 °C and 363 °C, respectively. In the propane atmosphere, the light-off temperature of the fresh Pd/6CeZrPAl catalyst was 20 °C lower than that of the Pd/CeZrAl catalyst, and the complete conversion temperature was 81 °C lower. The light-off temperature of the aged Pd/6CeZrPAl catalyst was 5 °C lower than that of the Pd/CeZrAl catalyst, and the complete conversion temperature was 15 °C lower. From the conversion curve, it can be seen that the Pd/6CeZrPAl catalyst has a significant advantage in terms of conversion rate compared to Pd/CeZrAl. The results are consistent with the propane oxidation catalyzed by a P-modified Pd/Al_2_O_3_-TiO_2_ catalyst [46]. The results indicate that the addition of P additive improves the catalyst activity. This may be attributed to the change of the structure, redox performance, and surface acidity of the catalyst after the addition of P [52].

Interestingly, it was found that the activity of the aged catalyst was better than that of the fresh catalyst, which was also found in our previous study on CH_4_ oxidation [53]. It has been reported that suitable Pd^2+^/(Pd^2+^ + Pd^0^) and Pd particle sizes on the surface of a catalyst are more conducive to the breakage of C-H bonds [24]. As reported, the existence state of Pd in the catalyst was determined through XPS with Pd^2+^and Pd^0^, and more metal Pd was formed, while the size of Pd particles also increased during the aging process. The supporting data for this result are reported in a previous study [25]. In addition, Table S5 also presents the data on the Pd particle size of catalysts. From the table, it can be seen that the particle size of Pd in the fresh catalyst is around 5.5 nm, while the particle size of Pd in the aged catalyst is around 12.4 nm. Compared with fresh catalysts, the particle size of Pd in aged catalysts increased. Garcia et al. [54] reported that the activity of the catalyst in propane oxidation could be improved when the particle size of Pd increased, and one of the reasons for the excellent activity of catalysts is the presence of large Pd particles. Moreover, studies on the activity of Pd catalysts loaded with different amounts of P on CH_4_ [53] and C_3_H_8_ [55] oxidation have been reported; the results showed that the addition of P is helpful for the activity of catalysts. In addition, this study focused on the effect of P addition on support properties. Therefore, no further exploration was conducted on the oxidation of C_3_H_8_ under different P contents of loaded Pd. As we know, catalysts lower the activation energy of reactions, thereby reducing the reaction barrier and increasing the number of activated molecules in chemical reactions. For propane oxidation, C-H cleavage is rate-determining [56,57]. A faster reaction rate means more activated molecules and a stronger ability to break the C–H bonds in propane. Liang et al. [46] studied the activation energy of Pd/AT and Pd/ATP under conditions of propane conversion below 15%. The study found that the activation energy of the catalyst with P added was significantly lower than that of the catalyst without P. Therefore, the addition of P reduces the activation energy of the chemical reaction and accelerates it, which has also been observed in Pd-based catalysts for the catalytic combustion reactions of CH_4_ [13] and C_3_H_6_ [55].

In reactions catalyzed by Pd-based catalysts, the presence of H_2_O usually accelerates the sintering of the metal and the deactivation of the support, and it forms inactive Pd(OH)_2_ species, which reduce the catalytic activity [1]. Numerous studies have been reported on the catalytic activity of catalysts, which have shown that the catalytic ability is essential, on the one hand, with the properties of the support, and, on the other hand, with the state of the active substance. Noronha et al. [3] reported that in the reaction of C_3_H_8_ oxidation, the redox process of surface Pd^0^ and PdO is a prerequisite for active catalysts, but the surface ratio of Pd^0^/Pd^2+^ may be a decisive factor in the observed catalytic behavior.

From the XRD and XPS results, we can infer that when P is added to CeZrAl, stable Ce (III) phosphate forms, and as the amount of P added increases, so does the amount of Ce (III) phosphate. And the properties of Ce (III) phosphate are very stable, leading to the inability of Ce^3+^ in CePO_4_ to form oxygen vacancies, resulting in a de-crease in the number of oxygen vacancies in the support and a decrease in the surface-adsorbed oxygen content. This is consistent with the OSC results. This, in turn, reduces the number of PdO formed, affecting the number of active species in the catalyst. Excess oxygen leads to the formation of too much Pd^2+^, while too little oxygen results in too much Pd^0^ in the reaction. With the increase in the amount of P added, the amount of Pd in the reaction gradually increases, with Pd^2+^ gradually decreasing, causing the Pd^0^/Pd^2+^ surface ratio to increase first and then decrease, reaching its optimal value at a P addition content of 6 wt.%. This is consistent with the experimental results in this study. The same phenomenon occurs in the Pd-catalyzed CH_4_ oxidation and C_3_H_8_ oxidation [46], which may be attributed to the effective promotion of the distribution of active Pd species and the regulation of the surface Pd^2+^/(Pd^2+^ + Pd^0^) ratio through the addition of P, synergistically promoting the oxidation of C_3_H_8_ [13,24].

Additionally, after adding P, the support CeZrAl forms samples containing P, which results in many micro-pores during the calcination process due to the decomposition of ammonium salts. The number of micro-pores gradually increases with the increase in the amount of P added. The small pores gradually grow into large pores when high-temperature aging occurs. Therefore, compared to the support without the addition of P, the support with the addition of P has a more stable texture. Compared with the aged CeZrAl sample, the specific surface area of the aged 6CeZrPAl sample slightly increases, indicating that 6CeZrPAl can provide a larger specific surface area and expose more active sites, thus producing a stronger catalytic effect. In summary, studying the support is of great significance for improving the activity of the catalyst.

## 4. Conclusions

We studied the effect of adding additive P on the properties of supports and the activity of catalysts prepared using it as a support. First, we studied the influence of preparation methods on the support. The results showed that the support prepared using the sol–gel method had a more stable phase structure, more oxygen species, and a larger oxygen storage capacity than other preparation methods. This is mainly because Al^3+^ in the support material prepared with the sol–gel method would enter the Ce_x_Zr_1−x_O_2_ solid solution lattice and cause crystal distortion, resulting in more oxygen vacancies and lattice defects in the Ce_x_Zr_1−x_O_2_ solid solution. The result is the generation of more adsorbed oxygen species, which can adsorb more oxygen and further enhance the oxygen storage capacity. Furthermore, the effect of the addition of P on the properties of the support material was studied, and the results showed that doping an appropriate amount of phosphorus can improve the specific surface area and stability of CeZrPAl composite oxides. This is mainly because the crystal growth of CePO_4_ in the composite material will reduce the Ce content in the cubic cerium zirconium solid solution, leading to the phase separation of the cerium zirconium solid solution. Furthermore, CePO_4_ could lock in some Ce^3+^/Ce^4+^ redox pairs, resulting in fewer oxygen species and a lower oxygen storage capacity of CeZrPAl composite oxides at higher phosphorus contents. Finally, the oxidation ability of the catalyst prepared by loading Pd onto the support material for C_3_H_8_ was investigated. The results showed that the support had better catalytic activity compared to the carrier without a P addition, as the addition of P improved the performance of the carrier. The study of catalyst support is of great significance for improving the activity, stability, and reusability of catalysts, as well as for developing new catalysts, and so on. This study will promote the development of catalysis in areas such as energy, environment, and chemical engineering.

## Figures and Tables

**Table 1:** XPS data of CeZrPAl composite oxides prepared using different methods.

Samples	Ce^3+^/(Ce^3+^ + Ce^4+^)	O_ads_/(O_latt_ + O_ads_ + O_OH_)
	(%)	(%)
CeZrPAl-G-A	36.7	54.4
CeZrPAl-I-A	41.4	52.0
CeZrPAl-M-A	30.3	49.6

**Table 2:** The specific surface area and pore structure information summary of CeZrPAl composite oxides prepared using different methods.

Samples	Specific Surface Area	Total Pore Volume	Pore Size
	(m^2^·g^−1^)	(cm^3^·g^−1^)	(nm)
CeZrPAl-G-F	323	0.41	0–17
CeZrPAl-I-F	217	0.29	0–16
CeZrPAl-M-F	255	0.35	0–16
CeZrPAl-G-A	88	0.29	0–98
CeZrPAl-I-A	69	0.23	0–100
CeZrPAl-M-A	95	0.27	0–100

**Table 3:** The oxygen storage capacity (OSC) of CeZrPAl composite oxides prepared using different methods.

Samples	OSC (μmol/g)	
	F	A
CeZrPAl-G	294	56
CeZrPAl-I	306	50
CeZrPAl-M	212	37

**Table 4:** The XPS data of CeZrPAl composite oxides with different P contents.

Samples	Ce^3+^/(Ce^3+^ + Ce^4+^)	O_ads_/(O_latt_ + O_ads_ + O_OH_)
	(%)	(%)
CeZrAl	27	58.1
2CeZrPAl	27.1	56.2
6CeZrPAl	36.7	54.4
10CeZrPAl	38.8	53.8

**Table 5:** Summary of the specific surface area and pore structure information of CeZrPAl composite oxides with different P contents.

Samples	State	Specific Surface Area	Total Pore Volume
		(m^2^·g^−1^)	(cm^3^·g^−1^)
CeZrAl	F	209	0.32
2CeZrPAl		239	0.35
6CeZrPAl		329	0.41
10CeZrPAl		341	0.47
CeZrAl	A	87	0.26
2CeZrPAl		75	0.31
6CeZrPAl		89	0.29
10CeZrPAl		80	0.24

**Table 6:** The OSC information of CeZrPAl composite oxides with different P contents.

Samples	OSC (μmol/g)	
	F	A
CeZrAl	313	125
2CeZrPAl	306	81
6CeZrPAl	294	56
10CeZrPAl	231	63

## Data Availability

Data are contained within the article.

## Supplementary Materials

The following supporting information can be downloaded at: https://www.mdpi.com/article/10.3390/ma17051003/s1, Figure S1: Raman spectra of the aged CeZrPAl composite oxides prepared using different methods; Figure S2: Raman spectra of the aged CeZrPAl composite oxides with different phosphorus contents; Table S1: The P content on the surface of the support prepared using different preparation (results presented in weight percentage) and mapping data based on the EDS; Table S2: The data of OSC of supports prepared using different methods based on normalized surface area data; Table S3: The P content on the surface of supports with different P content additions (results presented in weight percentage) and mapping data based on the EDS; Table S4: The data of OSC of supports with different P content additions based on normalized surface area data. Table S5: The data on Pd particle size of catalysts loaded on supports with and without P.
