# Supplementary material for: The Effect P Additive on the CeZrAl Support Properties and the Activity of the Pd Catalysts in Propane Oxidation
Source: Materials (Basel). 2024 Feb 22;17(5):1003. doi: 10.3390/ma17051003 (PMC10934912; doi:10.3390/ma17051003)
Supplement: Supplementary file 1 [file materials-17-01003-s001.zip › materials-2836372-supplementary.pdf]

Supporting Information (SI)

The Effect P Additive on the CeZrAl Support Properties and the Activity of the Pd Catalysts in Propane Oxidation

Feng Feng [1,2], Hong Li [3,4], Xingxia Yang [2,3,4,*], Chengxiong Wang [2,3,4], Yunkun Zhao [2,3,4], Hua Wang [1] and Junchen Du 2,3,4,*

[1] Faculty of Metallurgical and Energy Engineering, Kunming University of Science and Technology, Kunming 650093, China

[2] State-Local Joint Engineering Research Center of Precious Metal Catalytic Technology and Application, Kunming Sino-Platinum Metals Catalysts Co. Ltd., Kunming 650106, China

[3] Yunnan Precious Metal Laboratory Co., Ltd., Kunming 650100, China

[4] State Key Laboratory of Advanced Technologies for Comprehensive Utilization of Platinum Metals, Kunming Institute of Precious Metals, Kunming 650100, China

* Correspondence: xingxia.yang@spmcatalyst.com (X.Y.); dujunchen2006126@126.com (J.D.)

Table of contents

Figure S1. Raman spectra of the aged CeZrPAl composite oxides prepared by different methods **2**

Table S1. The P content on the surface of the support prepared by different preparation (results presented in weight percentage), mapping data based on the EDS **3**

Table S2. The data of OSC of supports prepared by different methods based on normalized surface area data **4**

Figure S2. Raman spectra of the aged CeZrPAl composite oxides with different phosphorus contents **5**

Table S3. The P content on the surface of supports with different P adding (results presented in weight percentage), mapping data based on the EDS **6**

Table S4. The data of OSC of supports with different P adding based on normalized surface area data **7**

Table S5 The data on Pd particle size of catalysts loaded on supports with and without P **8**

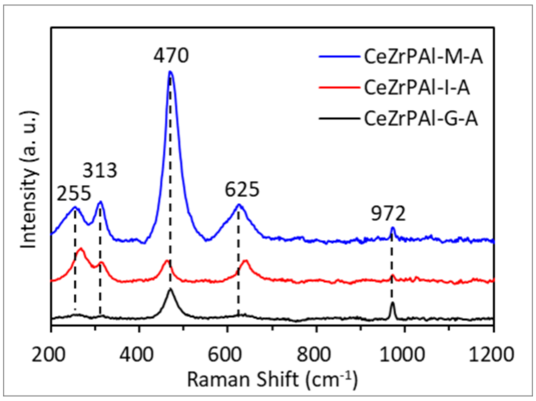

Figure S1. Raman spectra of the aged CeZrPAl composite oxides prepared by different methods.

Table S1 The P content on the surface of the support prepared by different methods (results presented in weight percentage), mapping data based on the EDS.

**Samples**	**P content (wt%)**	
	F	A
CeZrPAl-G	1.62	1.31
CeZrPAl-I	1.39	1.24
CeZrPAl-M	1.42	1.29

Table S2 The data of OSC of supports prepared by different methods based on normalized surface area data.

**Samples**	**OSC/SA (mmol/m²)**	
	F	A
CeZrPAl-G	0.91	0.63
CeZrPAl-I	1.41	0.72
CeZrPAl-M	0.83	0.39

Notes: Given as surface area, SA.

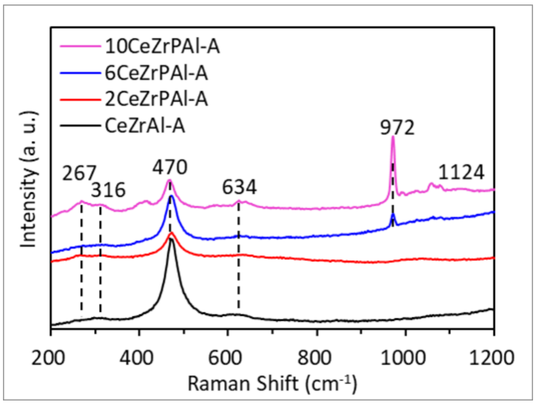

Figure S2. Raman spectra of the aged CeZrPAl composite oxides with different phosphorus contents.

Table S3 The P content on the surface of supports with different P adding (results presented in weight percentage), mapping data based on the EDS.

**Samples**	**P content (wt%)**	
	F	A
CeZrAl	0	0
2CeZrPAl	0	0
6CeZrPAl	1.62	1.31
10CeZrPAl	2.72	2.25

Table S4 The data of OSC of supports with different P adding based on normalized surface area data.

Samples	OSC/SA (mmol/m^2)	
	F	A
CeZrAl	1.50	1.44
2CeZrPAl	1.28	1.08
6CeZrPAl	0.89	0.63
10CeZrPAl	0.68	0.79

Notes: Given as surface area, SA.

Table S5 The data on Pd particle size of catalysts loaded on supports with and without P.

**Samples**	**State**	**Pd particle size （nm）**
CeZrAl	F	6.2
6CeZrPAl		4.9
CeZrAl	A	11.8
6CeZrPAl		13.0
